# Effect of ethylene-vinyl acetate and rubber waste on asphalt concrete performance

**DOI:** 10.3389/fchem.2025.1701958

**Published:** 2026-01-07

**Authors:** Saltanat Ashimova, Gulzat Aitkaliyeva, Nesipkhan Bektenov, Dinmukhambet Alizhanov, Gulnara Abdyrakhmanova, Nurman Sarybayev, Ilyas Baidullayev, Pietro Calandra, Cesare Oliviero Rossi

**Affiliations:** 1 Faculty of Natural Science and Geography, Abai Kazakh National Pedagogical University, Almaty, Kazakhstan; 2 Department of Chemical and Biochemical Engineering, Satbayev University, Almaty, Kazakhstan; 3 Department of Transport Construction, Kazakh Automobile and Road Institute named after L.B. Goncharov, Almaty, Kazakhstan; 4 JSC «Kazakhstan Road Research Institute», Astana, Kazakhstan; 5 CNR-ISMN, National Research Council, Institute for the Study of Nanostructured Materials, Montelibretti, Italy; 6 Department of Chemistry and chemical technologies, University of Calabria, Rende, Italy

**Keywords:** asphalt concrete, crumb rubber, ethylene-vinyl acetate (EVA), FTIR spectroscopy, polymer modification, rutting resistance, sustainable construction

## Abstract

The performance of asphalt concrete under increasing traffic loads and varying climatic conditions necessitates the development of enhanced bituminous binders. Some additives used as asphalt modifiers are polymeric materials. Examples of these polymers are styrene-butadiene rubber latex (SBR), diblock styrene-butadiene (SB) and triblock styrene-butadiene-styrene (SBS). The use of crumb rubber from worn-out tires can be considered as polymer-modified bitumen. This study investigates the effects of ethylene-vinyl acetate (EVA) granules and crumb rubber waste as modifiers on the physical, mechanical, and chemical properties of asphalt concrete. An ever-increasing pressure on waste resources and environmental protection leads to clear transition to a regenerative circular economy. The research aims to address the limitations of conventional bitumen, such as thermal instability and susceptibility to aging, particularly in regions with high traffic loads and extreme temperatures, by incorporating these polymer additives at 20% and 25% dosages relative to the binder mass. Laboratory-prepared asphalt concrete mixtures were evaluated for key performance indicators, including compressive strength at 0, 20 °C and 50 °C, water saturation, moisture resistance, crack resistance, shear stability, and rutting depth. Results demonstrated that EVA granules significantly improved thermal stability, with crack resistance at 0 °C doubling from 3.0 to 6.9 MPa. Compressive strength also increased to 2.2 MPa compared to the control sample (0.9 MPa). Rutting resistance was notably enhanced, with EVA-modified mixtures exhibiting an 85% reduction in rut depth (0.77 mm) compared to the unmodified mix (4.9 mm). Crumb rubber, while less effective in thermal performance, improved water resistance by reducing water saturation from 2.7% to 2.4% and demonstrated moderate gains in deformation resistance. Fourier-transform infrared spectroscopy (FTIR) revealed distinct chemical interactions between the modifiers and bitumen. EVA introduced polar functional groups (e.g., C=O at 1738 cm^-1^ and C-O-C at 1,242 cm^-1^), indicating chemical integration, whereas crumb rubber primarily influenced physical structure, evidenced by polyisoprene-related bands (966–970 cm^-1^). Economic analysis highlighted that EVA would be more cost-effective, due to lower material costs and superior performance. Both modifiers support sustainability by repurposing industrial waste. It turned out that both modifiers can contribute to environmental sustainability by repurposing industrial waste.

## 1 Introduction

The increasing volume of road traffic, heavier axle loads on pavements and extreme weather events have exposed the limitations of conventional asphalt pavements, particularly susceptibility to rutting, thermal cracking, and rapid aging (Emt et al., 2023; Rossi et al., 2017a), so that better performance of road construction materials are required.

Despite their widespread use and despite the relatively low cost, conventional unmodified bituminous binders exhibit limited thermal stability and narrow operational temperature (Zhang et al., 2024) and the poor resistance to oxidative degradation makes them prone to aging, leading to rutting at high temperatures and cracking at low temperatures (Ren et al., 2024). One of the most effective approaches to enhancing the durability of asphalt concrete is modifying bitumen with polymers (Rossi et al., 2017b), which improves its rheological properties, increases deformation resistance, and slows aging processes (Emt et al., 2023).

Among the most promising thermoplastic modifiers is ethylene-vinyl acetate (EVA), a copolymer of ethylene and vinyl acetate. Recent studies demonstrate that EVA-modified binders can reduce rutting by up to 80% while delaying oxidative aging due to their oxygen-scavenging capacity (Ren et al., 2024; Yan et al., 2021). In addition, it was found capable of forming a three-dimensional network structure within bitumen (Nazmey et al., 2024). This significantly increases stiffness and enhances the thermal resistance of the modified binder, thereby reducing pavement susceptibility to rutting (Janmohammadi et al., 2020; Nazmey et al., 2024). Thorough research provided foundational evidence that EVA increases binder stiffness and elasticity at high temperatures and low frequencies, depending on bitumen–polymer compatibility and EVA concentration (Airey, 2002). Findings from ref (Yan et al., 2022) confirm that both the concentration and molecular structure of EVA critically determine its effectiveness as a modifier. Moreover, the polar nature of EVA ensures good compatibility with bitumen and improves the material adhesive properties, also in the presence of water (Zhang et al., 2022). Another notable aspect of EVA is its potential in warm-mix applications due to its melt behavior, making it an attractive candidate for recycling-driven modification techniques (Yan et al., 2022). However, EVA-modified binders can become overly rigid at low temperatures, resulting in brittle behavior that limits their use in cold regions (Zhang et al., 2022).

To offset the drawbacks of EVA and achieve a balanced set of properties, composite modifiers—such as combinations with crumb rubber or bio-oil additives—are increasingly being explored (Ren et al., 2024).

In the context of growing attention to the environmental impact of construction, the reuse of waste materials, including polymeric and rubber-based waste, has become a key direction for sustainable development. This also to promote sustainability through waste valorization (Ban et al., 2025; Rossi et al., 2024).

For instance, a study (Rossi et al., 2024) demonstrated that leather, rubber, and plastic waste account for the major share of disposal costs and volumes. An ever-increasing pressure on waste resources and environmental protection leads to a clear transition to a regenerative circular economy (Aitkaliyeva et al., 2022; Alfè et al., 2022; Caputo et al., 2023). A large portion of these materials is still incinerated for energy recovery, whereas the adoption of reuse and recycling strategies could significantly reduce both environmental and economic burdens. Another study supports the viability of crumb rubber as a sustainable alternative to traditional modifiers (Xiang et al., 2009) through a preblending technique improving compatibility, mechanical performance, and aging resistance in modified asphalt.

Despite progress in bitumen polymer modification technologies, the effects of various secondary polymer additives—such as ground rubber waste and EVA granules—on the physico-mechanical properties of asphalt concrete remain insufficiently studied. This research addresses that gap by examining the effects of EVA and rubber waste on asphalt mixture properties.

In this context, Crumb rubber, derived from end-of-life tires, offers a complementary solution. Its viscoelasticity enhances crack resistance at low temperatures and dampens traffic-induced vibrations (Ban et al., 2025). Together with improving rutting resistance, EVA uniquely contributes to low-temperature flexibility, making it a balanced modifier for climate resilience (Ameri et al., 2012).

Yet, challenges persist, including incomplete compatibility with bitumen and higher production temperatures (Ren et al., 2024). Recent advances in hybrid modification (e.g., EVA-rubber blends) aim to balance these properties, though mechanistic understanding remains incomplete (Aitkaliyeva et al., 2024).

Specifically, this research investigates the influence of these modifiers on strength, deformation, and moisture-related performance indicators, including water absorption, water resistance, compressive strength, crack resistance, and shear stability. By combining rheological testing, FTIR spectroscopy, and lifecycle cost analysis, the study aims to provide a more comprehensive understanding of how secondary polymer modifiers influence the behavior of asphalt concrete, providing a roadmap for optimizing polymer-modified asphalt mixtures in diverse climates. The work is notable. The construction sector generates 1.3 billion tons of polymer waste annually, with tire rubber and EVA-based packaging contributing significantly to landfill burdens (Ameri et al., 2012) so their incorporation into asphalt would lead to undiscussed economic and environmental benefits. For instance, every ton of crumb rubber used in asphalt diverts 300 passenger tires from landfills while cutting production costs by 15%–20% (Ban et al., 2025). This also would align with UN Sustainable Development Goals (SDGs 9, 11, and 12) by reducing raw material consumption and CO_2_ emissions (Aitkaliyeva et al., 2024).

## 2 Materials and methods

### 2.1 Materials

This study utilized road bitumen of grade BND 70/100. The main physical and mechanical properties of the bitumen used are presented in Table 1.

**TABLE 1:** Physical and mechanical properties of BND 70/100 bitumen.

Indicators	Standard value	Actual result
Penetration depth at 25 °C (0.1 mm)	0.1 mm	82
Softening point	°С	48
Ductility at 25 °C	Cm	150
Fraass breaking point	°С	−22

**FIGURE 1:**
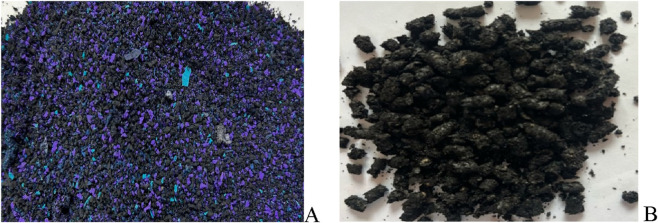
Appearance of the modifiers: **(A)** EVA granules; **(B)** crumb rubber.

Crumb rubber and EVA granules, shown in Figure 1, were used as industrial waste-based modifiers. To evaluate the effectiveness of these additives, a dry mixing method was applied. According to this method, the dosage of modifiers was set at 20% of the bitumen mass. This proportion was selected based on previous studies by other researchers (Lwin and Utomo, 2017).

The asphalt concrete type B mixtures with modifiers were prepared by weighing the calculated amount of raw materials, heating the aggregates in a drying oven to the required temperature (180 °C–185 °C), adding the modifiers and bitumen, and mixing the components in a laboratory paddle mixer. Mixing was carried out until a visually homogeneous blend was achieved.

### 2.2 Asphalt concrete preparation procedure

To prepare asphalt concrete of type B, in addition to bitumen and modifiers, the following mineral aggregates were used.

1. Crushed gravel aggregates (fractions 10–20 mm and 5–10 mm);
2. Sand from crushed stone screenings (fraction 0–5 mm);
3. Activated mineral filler MP-1.

All raw materials were tested for compliance with current standards. The particle size distributions of the gravel, sand, and mineral filler were determined. The physical and mechanical characteristics of the aggregates are presented in Tables 2–4.

**TABLE 2:** Physical and mechanical properties of crushed gravel aggregates.

Indicators	Standard value	Actual result	
		Aggregate size, mm	
		10–20	5–10
True density	g/cm^3^	2.70	2.70
Bulk density	g/cm^3^	2.60	2.59
Water absorption	%	0.8	0.8
Content of flaky particles	%	12	14
Content of dust and clay particles	%	0.1	0.14
Lump clay content	%	Not available	Not available
Crushability grade	​	1,000	1,000
Abrasion resistance grade	​	I-1	I-1
Frost resistance grade	​	F50	F50
Crushed particles content	%	91.2	90.7

**TABLE 3:** Physical and mechanical properties of sand from crushed stone screenings.

Indicators	Parameter	Actual result
True density	g/cm^3^	2.70
Bulk density	g/cm^3^	1.54
Crushability grade	​	1,000
Clay particle content (swelling method)	%	0.3

**TABLE 4:** Physical and mechanical characteristics of modified mineral powder MP-1.

Indicators	Parameter	Actual result
Grain size distribution −1.25 mm −0.315 mm −0.071 mm	%	100 99.6 90.2
Porosity	%	24.5
Swelling of specimens made from mineral powder–bitumen mixture	%	1.3
True density	g/cm^3^	2.7
Bulk density	g/cm^3^	2.04
Bitumen absorption capacity, g per 100 cm^3^ (absolute volume)	​	40.0
Moisture content	%	0.2

The crushed gravel aggregates are of high quality, exhibiting strong mechanical strength, high abrasion resistance, and adequate frost resistance for use in moderate climatic conditions. Low water absorption and a high percentage of crushed particles ensure good adhesion to the bituminous binder and contribute to the overall stability of the asphalt concrete mixture.

The sand obtained from crushed stone screenings demonstrates stable physical and mechanical properties. The true density is 2.70 g/cm^3^, indicating a typical mineral composition consistent with the gravel aggregates. The bulk density of 1.54 g/cm^3^ is acceptable for asphalt concrete mixtures, ensuring adequate compaction. A high crushability grade of 1,000 confirms the mechanical strength of the material. The clay particle content, determined by the swelling method, is 0.3%, which is within permissible limits and does not pose a significant risk to adhesion or moisture susceptibility.

At the second stage of the study, samples of type B asphalt concrete mixture were prepared in accordance with the requirements of standard ST RK 1225–2025, with a bitumen content of 5.0%. To evaluate the effect of modifying additives, a series of experimental mixtures was produced by incorporating 20% modifier by weight relative to the bitumen content. A reference mixture without any additives was used as the control sample. The distribution of mineral components in the mixture is presented in Table 5.

**TABLE 5:** Aggregate content.

Type of material	Content, %
Crushed stone, 15–20 mm fraction	13
Crushed stone, 10–15 mm fraction	12
Crushed stone, 5–10 mm fraction	20
Crushed stone screenings, 0–5 mm fraction	48
Activated mineral filler	7

### 2.3 Methods for studying the physical and mechanical properties of asphalt concrete

The physical and mechanical characteristics of asphalt concrete mixtures were investigated in accordance with the requirements of international ASTM standards. The samples were tested using the following indicators:

Water saturation was determined as the ratio of the mass of water absorbed by the sample to its dry mass, in accordance with ASTM D2726. Compressive strength was measured at temperatures of 20 °C and 50 °C in accordance with ST RK 1218. Moisture resistance under prolonged water saturation was assessed as the ratio of the strength of the water-saturated sample to the strength in a dry state, following ASTM D4867. Crack resistance was determined based on tensile strength at splitting at 0 °C and a deformation rate of 50 mm/min, in accordance with ASTM D6931. Shear stability was evaluated using the internal friction coefficient and shear adhesion measured at 50 °C.

The resistance of asphalt concrete mixtures to plastic deformation was assessed according to the European standard EN 12697–22. This method simulates cyclic vehicular loads on the specimen at 60 °C, enabling conditions close to real-life pavement operation in hot climates. The rut depth was measured after 10,000 wheel passes. Three types of type B asphalt concrete mixtures were prepared for the test: a control mixture (without modifier), a mixture with 25% rubber granulate, and a mixture with 25% EVA granules (based on the binder mass).

### 2.4 Study of the influence of modifying additives on the physicochemical properties of asphalt concrete

To assess the chemical changes occurring in bitumen as a result of modification, Fourier-transform infrared spectroscopy (FTIR) was employed. The analysis was carried out in the range of 4,000–400 cm^-1^ using an ALPHA II spectrometer.

## 3 Results and discussion

### 3.1 Comparative analysis of the physical and mechanical properties of asphalt concrete mixtures

This study presents a comprehensive evaluation of the effect of secondary polymer modifiers on key physical and mechanical properties of asphalt concrete mixtures. The aim of the analysis was to determine the degree of improvement in performance characteristics with the addition of EVA granules and rubber granulate, as well as to identify differences in their mechanisms of action.

Particular attention was paid to parameters such as compressive strength at different temperatures, water saturation, moisture resistance, crack resistance, shear stability, and rutting resistance. These indicators are critically important for assessing the durability and operational reliability of road pavements under variable climatic and mechanical loads.

Analysis of the control type B asphalt concrete mixture (Table 6) showed that the compressive strength at 50 °C was 0.9 MPa, and the shear adhesion was 0.21 MPa. These values indicate insufficient thermal stability and low resistance to plastic deformation, which may limit pavement durability in hot climates and under heavy traffic. Therefore, it is necessary to modify the mixture to enhance its performance characteristics in accordance with international road construction standards and methodologies.

**TABLE 6:** Physical and mechanical characteristics of unmodified asphalt concrete.

Indicators	Parameter	Actual result
Water saturation	%	2.7
Compressive strength at 20 °C	MPa	4.4
Compressive strength at 50 °C	MPa	0.9
Moisture resistance after prolonged water saturation	%	0.85
Shear resistance (internal friction coefficient) Shear adhesion at 50 °C	MPa	0.94 0.21
Crack resistance (tensile splitting strength at 0 °C and 50 mm/min deformation rate)	MPa	3.0

The introduction of secondary polymer additives had a significant effect on the properties of asphalt concrete mixtures. According to the data presented in Table 7, the use of rubber granulate led to a reduction in the water saturation of asphalt concrete from 2.7% to 2.4%, indicating increased density and structural homogeneity of the mixture. Similar observations are reported in (Ban et al., 2025), where it was shown that rubber particles enhance the contact between the binder and the mineral skeleton, contributing to a reduction in air voids and improved moisture resistance.

**TABLE 7:** Physical and mechanical characteristics of modified asphalt concrete.

No	Indicators	Actual results					
		Crumb rubber			EVA granules		
		10%	20%	25%	10%	20%	25%
1	Density, g/cm^3^	2.37	2.38	2.39	2.36	2.39	2.40
2	Water saturation, %	1.6	1.8	2.4	2.3	2.7	3.3
3	Compressive strength at 20 °C, MPa, not less than	4.1	4.5	4.7	3.1	3.2	3.4
4	Compressive strength at 50 °C, MPa, not less than	1.6	1.7	1.9	1.5	1.8	2.2
5	Compressive strength at 0 °C, MPa, not less than	6.5	8.6	9.3	7.4	8.4	9.8
6	Shear resistance based on - Internal friction coefficient, not less than - Shear adhesion at 50 °C, MPa, not less than	0.87 0.43	0.89 0.44	0.90 0.50	0.88 0.49	0.90 0.53	0.90 0.55
7	Crack resistance based on tensile strength at splitting at 0 °C and deformation rate of 50 mm/min, MPa, not less than	4.6	4.0	3.5	4.8	6.0	6.9
8	Rutting depth, mm, not more than	4.0	3.7	3.5	3.89	1.02	0.77

At the same time, the addition of EVA granules resulted in an increase in water saturation of the asphalt concrete to 3.3%. This may be attributed to the polar nature of the modifier itself, as well as its less effective adhesion to mineral components and bitumen. Moreover, when using the dry mixing method, EVA granules may distribute unevenly, causing local structural defects and increased porosity. Despite this, mechanical properties such as crack resistance and rutting resistance showed significant improvement, confirming the effectiveness of EVA as a modifying additive when the dosage and mixing technology are properly selected. For instance, the use of 25% EVA granules increased the thermal resistance of the mixture, doubling crack resistance at 0 °C doubling from 3.0 to 6.9 MPa. Compressive strength also increased to 2.2 MPa compared to the control sample (0.9 MPa).

Thus, modification with EVA granules contributes to the formation of more thermally stable and crack-resistant asphalt concrete. These results confirm the high efficiency of EVA as a modifier, provided that the optimal component ratio is maintained.

The resistance of asphalt mixtures to plastic deformation under prolonged loading is one of the key criteria for their operational reliability. This parameter is especially important in regions with hot climates and heavy traffic, where rutting significantly reduces the service life of the pavement.

**FIGURE 2:**
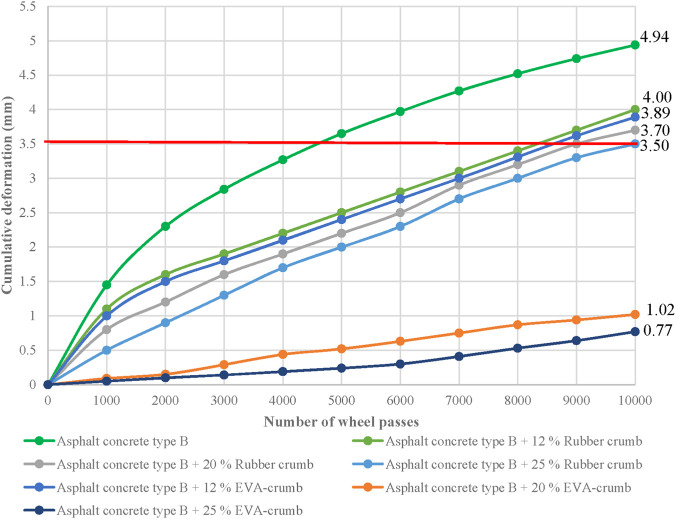
Rutting resistance of asphalt concrete.

As part of this study, three types of asphalt mixtures were analyzed: a control mixture (without modifiers), and compositions containing 25% EVA granules and rubber granulate. A comparative analysis of the results (Figure 2) allows assessment of the effectiveness of the modifiers in improving resistance to plastic deformation.

Analysis of the experimental data (Figure 2) shows a significant reduction in rut depth when polymer modifiers are used. In the control mix without additives, the residual deformation reached 4.9 mm after 10,000 wheel passes at a temperature of 60 °C, indicating high susceptibility to plastic flow. The addition of 25% rubber granulate reduced this value to 3.5 mm, reflecting a partial improvement in the structural stability of the pavement. The most pronounced effect was observed with EVA granule modification: the rut depth was only 0.77 mm, which represents an almost 85% reduction compared to the control mix.

These results confirm the high efficiency of EVA as a modifier capable of significantly enhancing the resistance of asphalt pavement to permanent deformation, due to the formation of a network structure within the bitumen matrix and improved rheological performance at elevated temperatures.

### 3.2 Study of the physico-chemical properties of unmodified and modified binders

FTIR spectroscopy has been chosen as an investigative tool because it can give information on the degrees of inter- and intra-molecular interactions in the systems under study.

**FIGURE 3:**
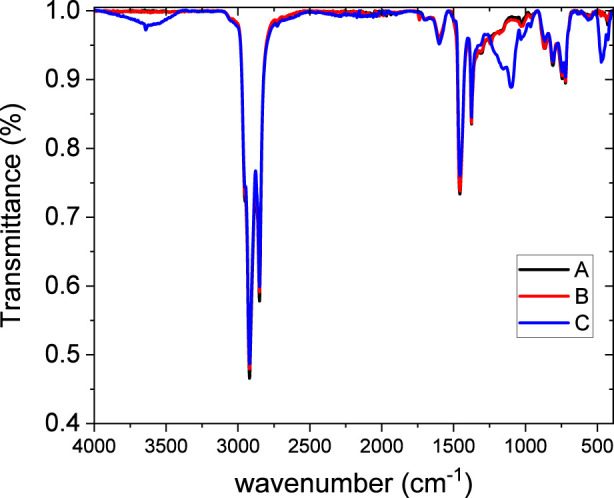
FTIR spectra of the unmodified bitumen (A), bitumen modified with EVA granules (B), and rubber crumb (C).

Figure 3 presents the FTIR spectra of the original asphalt concrete and its modified forms with EVA granules and rubber crumb. All spectra show characteristic absorption bands: stretching vibrations of C–H bonds in alkyl groups (∼2,920 and ∼2,850 cm^-1^), deformation vibrations of CH_2_/CH_3_ (∼1,455 cm^-1^) and CH_3_ (∼1,375 cm^-1^), as well as a band around 1,030 cm^-1^ associated with S=O group vibrations (Aitkaliyeva et al., 2025). Complete band attribution is reported in Table 8.

**TABLE 8:** Infrared frequencies of functional groups and their assignments.

Wavenumber (cm^-1^)	Group assignement
3,970–3,370 2,951 (not resolved) 2,920 2,867 (shoulder) 2,850 1730 1,455 1,375 1,242 1,030	OH and NH stretchings (bound molecules) CH_3_ antisymmetric stretching CH_2_ antisymmetric stretching CH_3_ symmetric stretching CH_2_ symmetric stretching C=O stretching Deformation vibrations of CH_2_/CH_3_ Deformation vibrations of CH_3_ C–O–C vibrations S=O stretching

**FIGURE 4:**
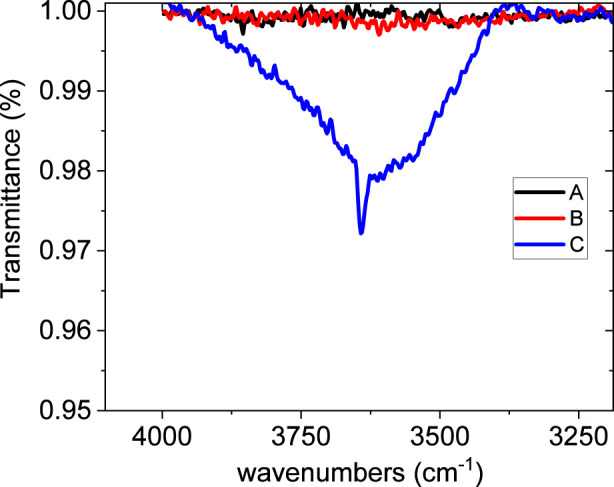
Magnified portion of the 3,250–4,000 cm^-1^ range of the FTIR spectra unmodified bitumen (A), bitumen modified with EVA granules (B), and rubber crumb (C).

It is worth noting the presence of a weak and broad band at the very high side frequency in the 4,000–3,350 cm^-1^ range. Magnified portion is reported in Figure 4.

This feature is generally due to the OH or NH functional groups in bound molecules, with a sharp contribution at 3,640 cm^-1^ emerging from the broad band and most probably due to the presence of some monomers (not interacting OH- or NH- containing molecules). While both O-H and N-H stretches appear in the general vicinity of 3,300 cm^-1^, the width and intensity of the peaks, along with other spectral data, can be used to distinguish between them. O-H stretches are generally broader and stronger, while N-H stretches are typically narrower and weaker, due to differences in hydrogen bonding and electronegativity (Pavia et al., 2014). This band feature is typical of crumb rubber due to its content in O and N atoms, and consequently the bitumen containing it exhibits the corresponding IR feature. However, in absence of further accompanying information it is hard to distinguish what kind of amine/alcohol is involved from the purely IR band detection.

**FIGURE 5:**
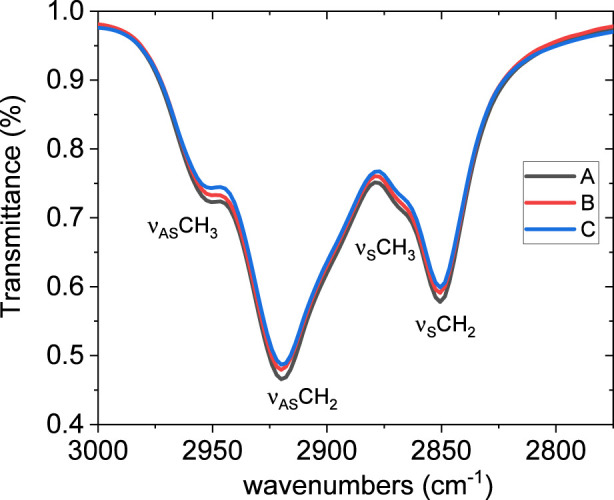
Portion of the FTIR spectra showing the symmetric and antisymmetric stretching of CH_2_ and CH_3_ groups unmodified bitumen (A), bitumen modified with EVA granules (B), and rubber crumb (C).

Perusal of the 3,000–2,800 cm^-1^ spectral range, which is reported in Figure 5, can give further interesting information. In this region the peaks are due to the symmetric and antisymmetric stretching (ν_S_ and ν_AS_ respectively) of CH_2_ and CH_3_ groups, as indicated in the figure as labels. Considering that these groups are located along the alkyl chain forming aliphatic portions of the molecules, most reasonably located in the maltene fraction of the system, the relative intensity ratio of such peaks is sensitive to the molecular lateral packing of the alkyl chains, as found in some model systems (Calandra et al., 2000).

It must be pointed out that an increase in the lateral packing order of the alkyl chains results in a decrease in the intensity of the CH_2_ symmetric stretching band relative to the CH_3_ antisymmetric stretching band (Cavallaro et al., 1995; Edwards et al., 1993).

It can be seen, here, that no difference in any of the peak positions takes place. This shows that a change in the lateral packing of the alkyl chains both in the skeletal CH_2_ groups and in the terminal CH_3_ groups is ruled out. Only some small deviations, just above from the statistical error, takes place in the intensity, but it affects almost in the same way all the four contributions, ruling also out changes in relative intensity among these absorptions. Consequently, the local structure experienced by the alkyl chains is not dependent on the presence of EVA or crumb rubber. This is an indication that the alkylic parts of the systems, most reasonable in the maltenic fraction is not affected by the presence or absence of crumb rubber or EVA granules, reasonably due to the scarce interactions with them. After all, the addition of such materials involves the insertion of bulk (granules) particles, with clearly negligible surface-to-volume ratio, and therefore the interactions of bituminous molecules with the hosted (EVA or crumb rubber) particles can involve only their surface, with the results that such interactions must be minimal. The fact that alyphatic parts of the molecules have inherently scarce chemical affinity and therefore low tendency to establish interactions with EVA and crumb rubber, completes the job.

The modification of asphalt concrete with rubber crumb is accompanied by the appearance or intensification of a band in the 966–970 cm^-1^ region, characteristic of vinyl fragments of polyisoprene, as well as in the intensity of bands in the 1,030–1,050 cm^-1^ range.

**FIGURE 6:**
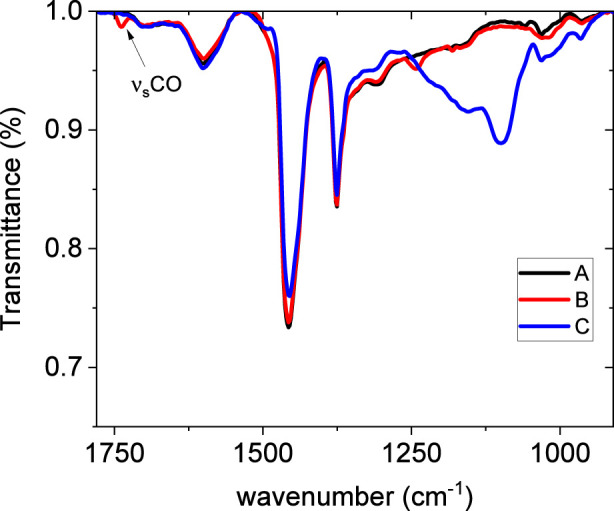
FTIR spectra (950–1,500 cm^-1^) of bitumen samples unmodified bitumen (A), bitumen modified with EVA granules (B), and rubber crumb (C).

This spectral portion is better shown by magnification in Figure 6 and the difference highlighted in crumb rubber containing bitumen may indicate interactions between the rubber and oxygen-containing groups in the asphalt concrete. At the same time, the pronounced bands at ∼1,455 cm^-1^ and ∼1,375 cm^-1^, due to the deformation vibrations of CH_2_/CH_3_ and CH_3_, respectively, shown in the same Figure 6, remains almost unaffected, reflecting the stability of the hydrocarbon backbone of the modified material.

The spectra of asphalt concrete modified with EVA show distinct changes: the appearance of a peak at 1,242 cm^-1^, associated with C–O–C vibrations (from ester groups), may correspond to the stretching vibration of C–OH bonds. This may indicate the formation of hydroxyl or ether functional groups as a result of interactions between EVA and the oxygen-containing components of the bituminous matrix, which is consistent with the findings reported in (Airey, 2002). Coherently, the peak at 1738 cm^-1^, due to the carbonyl functional group absorption, is clearly visible in the spectrum of EVA-containing bitumen, since it is characteristics of this polymer.

Thus, the FTIR spectra confirm the presence of specific functional groups associated with EVA and rubber crumb, as well as their chemical interaction with the asphalt concrete. EVA modification has a more pronounced effect on the chemical structure of the binder due to the introduction of polar groups, whereas rubber granules mainly affect the physical structure of the matrix.

### 3.3 Economic feasibility assessment of EVA and rubber granule application

To assess the feasibility of using secondary polymer modifiers, a techno-economic evaluation was conducted to estimate the impact of EVA granules and rubber crumb on the cost of asphalt concrete mixtures and the overall economics of road construction.

For this evaluation, the dosage of polymer modifiers was set at 25% by weight of the binder in the asphalt mixture, as this proportion provided the best performance in terms of strength, crack resistance, and deformation stability. This dosage was adopted as a reference point to estimate the effect of polymer additives on the overall cost of the pavement. The calculations are based on current market prices for the materials used, excluding costs associated with transportation, production, and laying. However, the enhanced performance characteristics achieved through modification are expected to reduce the need for routine and capital repairs, thereby reinforcing the overall economic viability of this approach.

As shown in Table 9, the greatest economic benefit is achieved when using EVA granules, which is attributed to their lower cost compared to crumb rubber, as well as a significant reduction in the overall cost of the asphalt mixture. The resulting savings can reach up to 8.55 USD/m^2^ compared to the unmodified mixture.

**TABLE 9:** Influence of modifier type on the cost of asphalt concrete pavement.

Indicators	Without modifier	Rubber granule	EVA granules
Modifier cost, USD/kg	-	1.48	0.83
Modifier dosage, % of binder mass	-	20	20
Total pavement cost, USD/m^2^	61.95	61.52	53.40
Cost savings compared to control mix, USD/m^2^	-	0.43	8.55
Environmental impact	Not applicable	However, the use of tire and EVA waste contributes to waste reduction and lowers disposal-related costs	

It should be noted that the presented calculations are based on market prices of materials and an assumed modifier dosage (25% by binder weight). The economic evaluation considered only the material costs, excluding transportation, paving, and maintenance expenditures. Nevertheless, the improvement in performance characteristics of the modified mixtures can potentially reduce the frequency and volume of repair operations, further reinforcing the economic feasibility of the proposed approach.

## 4 Conclusion

This study presented a comprehensive evaluation of the impact of secondary polymer modifiers—EVA granules and crumb rubber—on the performance of type B asphalt concrete mixtures. The results demonstrated that both modifiers enhance the mechanical and durability properties of the material, although the degree of improvement varied significantly.

The addition of EVA granules led to a notable increase in rutting resistance (reducing rut depth to 0.77 mm), improved cracking resistance (up to 6.9 MPa), and enhanced compressive strength at elevated temperatures. Crumb rubber also showed a positive effect, particularly in improving water resistance, although its overall impact was less pronounced compared to EVA. FTIR spectroscopy confirmed the different modification mechanisms, highlighting the structural activity of EVA and the primarily physical influence of rubber granules.

Furthermore, the techno-economic analysis showed that the use of EVA granules offers the highest cost savings (up to 8.55 USD/m^2^) while simultaneously reducing environmental impact through the recycling of polymer waste.

In conclusion, the use of EVA granules as a bitumen modifier represents an effective and environmentally sustainable approach to enhancing the reliability and longevity of asphalt pavements. Future research should focus on assessing the performance of the modified mixtures under low-temperature conditions, evaluating the influence of climatic factors, and conducting large-scale field tests to validate the laboratory findings under real-world conditions.

## Data Availability

The datasets are available by request to Prof Cesare Oliviero Rossi, second corresponding author. Requests to access the datasets should be directed to cesare.oliviero@unical.it.
