# Correction: Effect of ethylene-vinyl acetate and rubber waste on asphalt concrete performance

**DOI:** 10.3389/fchem.2026.1808130

**Published:** 2026-03-30

**Authors:** Saltanat Ashimova, Gulzat Aitkaliyeva, Nesipkhan Bektenov, Dinmukhambet Alizhanov, Gulnara Abdyrakhmanova, Nurman Sarybayev, Ilyas Baidullayev, Pietro Calandra, Cesare Oliviero Rossi

**Affiliations:** 1 Faculty of Natural Science and Geography, Abai Kazakh National Pedagogical University, Almaty, Kazakhstan; 2 Department of Chemical and Biochemical Engineering, Satbayev University, Almaty, Kazakhstan; 3 Department of Transport Construction, Kazakh Automobile and Road Institute named after L.B. Goncharov, Almaty, Kazakhstan; 4 JSC Kazakhstan Road Research Institute, Astana, Kazakhstan; 5 CNR-ISMN, National Research Council, Institute for the Study of Nanostructured Materials, Montelibretti, Italy; 6 Department of Chemistry and chemical technologies, University of Calabria, Rende, Italy

**Keywords:** asphalt concrete, crumb rubber, ethylene-vinyl acetate (EVA), FTIR spectroscopy, polymer modification, rutting resistance, sustainable construction

An incorrect Funding statement was provided. The correct funding statement reads:

The author(s) declared that financial support was received for this work and/or its publication. This research was funded by Consorzio Interuniversitario Nazionale per la Scienza e Tecnologia dei Materiali – INSTM, Italy.

The original article has been updated.

## Publisher’s note

All claims expressed in this article are solely those of the authors and do not necessarily represent those of their affiliated organizations, or those of the publisher, the editors and the reviewers. Any product that may be evaluated in this article, or claim that may be made by its manufacturer, is not guaranteed or endorsed by the publisher.

